# Supplementary material for: Hematological and physiological responses in polo ponies with different field-play positions during low-goal polo matches
Source: PLoS One. 2024 May 16;19(5):e0303092. doi: 10.1371/journal.pone.0303092 (PMC11098493; doi:10.1371/journal.pone.0303092)
Supplement: S1 Fig — In addition, this file also includes the HR time series and raw RR interval data. (DOCX) [file pone.0303092.s001.docx]

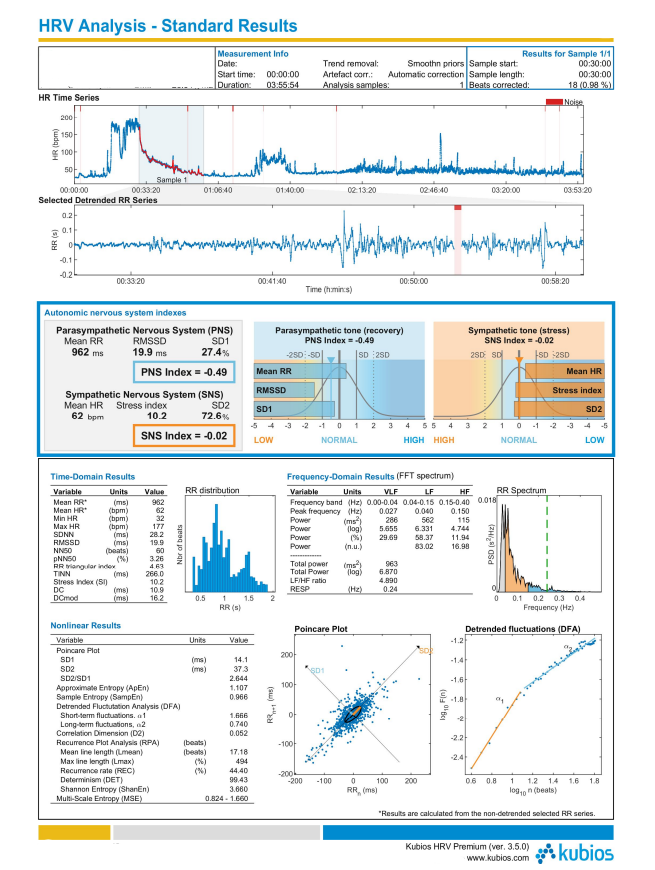


**Fig S1.** The MATLAB MAT-file demonstrates all analysis results of a polo pony participating in the match play, including time-domain result, frequency-domain results nonlinear results and autonomic nervous system indexes. In addition, this file also includes the HR time series and raw RR interval data.
