# Supplementary material for: Hematological and physiological responses in polo ponies with different field-play positions during low-goal polo matches
Source: PLoS One. 2024 May 16;19(5):e0303092. doi: 10.1371/journal.pone.0303092 (PMC11098493; doi:10.1371/journal.pone.0303092)
Supplement: Attachment — Submitted filename: PONE_D_23_41528_Reviewed.pdf [file pone.0303092.s002.pdf]

PLOS ONE

Hematology, physiology, and autonomic regulation in polo ponies with different field-play positions during low-goal polo matches

--Manuscript Draft--

Manuscript Number: PONE-D-23-41528

Article Type: Research Article

Full Title: Hematology, physiology, and autonomic regulation in polo ponies with different field-play positions during low-goal polo matches

Short Title: Physiological and autonomic responses in polo ponies with different field-play positions

Corresponding Author: Metha Chanda, D.V.M.,Ph.D., DTBVM
Kasetsart University Faculty of Veterinary Medicine Kamphaengsaen Campus
Nakorn Pathom, Nakorn Pathom THAILAND

Keywords: effort intensity; polo; field-play position; horse; physiology; stress

Abstract: This study investigated effort intensity and the impact of exertion on blood cortisol, hematology, and heart rate variability (HRV) in polo ponies with different field-play positions. Thirty-two ponies were divided equally into eight teams, each comprising forwarders (number 1), midfielders (numbers 2 and 3), and defenders (number 4). On consecutive days, the team pairs played the first chukka in four low-goal polo matches. Percent maximum heart rate (%HRmax), indicating ponies' effort intensity, was classified into five zones, including zones 1 (<70%), 2 (70–80%), 3 (80–90%), 4 (90–95%) and 5 (>95%). Blood parameters were determined before, immediately after, and at 30-minute intervals for 180 minutes after chukkas. HRV variables were evaluated before, during warm-up and exercise periods, and 30-minute intervals for 180 minutes after chukkas. Results indicated that number two ponies spent more time in zone 4 ($p < 0.05$) but less in zone 2 ($p < 0.01$) than the number four ponies. Cortisol levels increased immediately and 30 minutes afterward ($p < 0.0001$ for both periods) and then returned to baseline 60–90 minutes after exertion. Other measures (Hct, Hb, RBC, WBC, neutrophils, and CK enzyme) increased immediately ($p < 0.0001$ for all variables) and lasted at least 180 minutes after exertion ($p < 0.05$–0.0001). HRV decreased during the chukka until approximately 90 minutes afterward ($p < 0.05$–0.0001). The stress index increased during the chukka and declined to baseline at 60 minutes in number 1–3 ponies but lasted 90 minutes in those at number four. Effort intensity distribution differed among field-play positions. Decreased HRV indicated reduced parasympathetic activity, corresponding to increased sympathetic activity during warm-up and workout, extending to 90 minutes after exertion. Defenders seem to experience more stress than those in other positions.

Order of Authors:
Kanokpan Sanigavatee, D.V.M.
Chanoknun Poochipakorn, D.V.M., MS.
Onjira Huangsaksri, D.V.M.
Thita Wonghanchao, D.V.M.
Mona Yalong, D.V.M.
Kanoknoot Poungpuk, D.V.M.
Kemika Thanaudom, D.V.M.
Metha Chanda, D.V.M.,Ph.D., DTBVM

Additional Information:

Question	Response
**Financial Disclosure**<br><br>Enter a financial disclosure statement that describes the sources of funding for the	The author(s) received no specific funding for this work.

Powered by Editorial Manager® and ProduXion Manager® from Aries Systems Corporation

work included in this submission. Review the submission guidelines for detailed requirements. View published research articles from *PLOS ONE* for specific examples.<br><br>This statement is required for submission and **will appear in the published article** if the submission is accepted. Please make sure it is accurate.<br><br>**Funded studies**<br>Enter a statement with the following details:<br>• Initials of the authors who received each award<br>• Grant numbers awarded to each author<br>• The full name of each funder<br>• URL of each funder website<br>• Did the sponsors or funders play any role in the study design, data collection and analysis, decision to publish, or preparation of the manuscript?<br><br>Did you receive funding for this work?	
**Competing Interests**<br><br>Use the instructions below to enter a competing interest statement for this submission. On behalf of all authors, disclose any competing interests that could be perceived to bias this work—acknowledging all financial support and any other relevant financial or non-financial competing interests.<br><br>This statement is required for submission and **will appear in the published article** if the submission is accepted. Please make sure it is accurate and that any funding sources listed in your Funding Information later in the submission form are also declared in your Financial Disclosure statement.<br><br>View published research articles from *PLOS ONE* for specific examples.	The authors have declared that no competing interests exist.

Powered by Editorial Manager® and ProduXion Manager® from Aries Systems Corporation

**NO authors have competing interests**<br><br>Enter: *The authors have declared that no competing interests exist.*<br><br>**Authors with competing interests**<br><br>Enter competing interest details beginning with this statement:<br><br>*I have read the journal's policy and the authors of this manuscript have the following competing interests: [insert competing interests here]*<br><br>* typeset	
**Ethics Statement**<br><br>Enter an ethics statement for this submission. This statement is required if the study involved:<br><br>• Human participants<br>• Human specimens or tissue<br>• Vertebrate animals or cephalopods<br>• Vertebrate embryos or tissues<br>• Field research<br><br>Write "N/A" if the submission does not require an ethics statement.<br><br>General guidance is provided below. Consult the submission guidelines for detailed instructions. **Make sure that all information entered here is included in the Methods section of the manuscript.**	The study protocol was approved by the Kasetsart University's Institute of Animal Care and Use Committee (ACKU65-VET-003).

Powered by Editorial Manager® and ProduXion Manager® from Aries Systems Corporation

**Format for specific study types**<br><br>**Human Subject Research (involving human participants and/or tissue)**<br>• Give the name of the institutional review board or ethics committee that approved the study<br>• Include the approval number and/or a statement indicating approval of this research<br>• Indicate the form of consent obtained (written/oral) or the reason that consent was not obtained (e.g. the data were analyzed anonymously)<br><br>**Animal Research (involving vertebrate animals, embryos or tissues)**<br>• Provide the name of the Institutional Animal Care and Use Committee (IACUC) or other relevant ethics board that reviewed the study protocol, and indicate whether they approved this research or granted a formal waiver of ethical approval<br>• Include an approval number if one was obtained<br>• If the study involved *non-human primates*, add *additional details* about animal welfare and steps taken to ameliorate suffering<br>• If anesthesia, euthanasia, or any kind of animal sacrifice is part of the study, include briefly which substances and/or methods were applied<br><br>**Field Research**<br><br>Include the following details if this study involves the collection of plant, animal, or other materials from a natural setting:<br>• Field permit number<br>• Name of the institution or relevant body that granted permission	
**Data Availability**<br><br>Authors are required to make all data underlying the findings described fully available, without restriction, and from the time of publication. PLOS allows rare exceptions to address legal and ethical concerns. See the PLOS Data Policy and FAQ for detailed information.	Yes - all data are fully available without restriction

A Data Availability Statement describing where the data can be found is required at submission. Your answers to this question constitute the Data Availability Statement and **will be published in the article**, if accepted.<br><br>**Important:** Stating 'data available on request from the author' is not sufficient. If your data are only available upon request, select 'No' for the first question and explain your exceptional situation in the text box.<br><br>Do the authors confirm that all data underlying the findings described in their manuscript are fully available without restriction?	
**Describe where the data may be found in full sentences. If you are copying our sample text, replace any instances of XXX with the appropriate details.**<br><br>• If the data are **held or will be held in a public repository**, include URLs, accession numbers or DOIs. If this information will only be available after acceptance, indicate this by ticking the box below. For example: *All XXX files are available from the XXX database (accession number(s) XXX, XXX.).*<br>• If the data are all contained **within the manuscript and/or Supporting Information files**, enter the following: *All relevant data are within the manuscript and its Supporting Information files.*<br>• If neither of these applies but you are able to provide **details of access elsewhere**, with or without limitations, please do so. For example:<br><br>*Data cannot be shared publicly because of [XXX]. Data are available from the XXX Institutional Data Access / Ethics Committee (contact via XXX) for researchers who meet the criteria for access to confidential data.*<br><br>*The data underlying the results presented in the study are available from (include the name of the third party*	The HRV data supporting this study's findings are available at https://doi.org/10.6084/m9.figshare.24785247.v1

Powered by Editorial Manager® and ProduXion Manager® from Aries Systems Corporation

and contact information or URL).<br>• This text is appropriate if the data are owned by a third party and authors do not have permission to share the data.<br><br>* typeset	
Additional data availability information:	Tick here if the URLs/accession numbers/DOIs will be available only after acceptance of the manuscript for publication so that we can ensure their inclusion before publication.

Powered by Editorial Manager® and ProduXion Manager® from Aries Systems Corporation

Manuscript

Click here to access/download;Manuscript;Main text polo (English proofread).docx

Hematology, physiology, and autonomic regulation in polo ponies with different field-play positions during low-goal polo matches

Running head: Physiological and autonomic responses in polo ponies with different field-play positions

Kanokpan Sanigavatee[1,3¶], Chanoknun Poochipakorn[1,3¶], Onjira Huangsaksri[1,3¶], Thita Wonghanchao[1,3¶], Mona Yalong[2&], Kanoknoot Poungpuk[2&], Kemika Thanaudom[2&] and Metha Chanda[3,4*]

[1] Veterinary Clinical Study Program, Faculty of Veterinary Medicine, Kasetsart University, Kampaeng Saen Campus, Nakorn Pathom 73140, Thailand

[2] Veterinary Science Program, Faculty of Veterinary Medicine, Kasetsart University, Kampaeng Saen Campus, Nakorn Pathom 73140, Thailand

[3] Department of Large Animal and Wildlife Clinical Science, Faculty of Veterinary Medicine, Kasetsart University, Kampaeng Saen Campus, Nakorn Pathom 73140, Thailand

[4] Thailand Equestrian Federation, Sports Authority of Thailand, Bangkok 10330, Thailand

* Corresponding author

E-mail: fvetmtcd@ku.ac.th (MC)

[¶] These authors contributed equally to this work.

[&] These authors also contributed equally to this work.

Abstract

This study investigated effort intensity and the impact of exertion on blood cortisol, hematology, and heart rate variability (HRV) in polo ponies with different field-play positions. Thirty-two ponies were divided equally into eight teams, each comprising forwarders (number 1), midfielders (numbers 2 and 3), and defenders (number 4). On consecutive days, the team pairs played the first chukka in four low-goal polo matches. Percent maximum heart rate (%HR_{max}), indicating ponies' effort intensity, was classified into five zones, including zones 1 (<70%), 2 (70–80%), 3 (80–90%), 4 (90–95%) and 5 (>95%). Blood parameters were determined before, immediately after, and at 30-minute intervals for 180 minutes after chukkas. HRV variables were evaluated before, during warm-up and exercise periods, and 30-minute intervals for 180 minutes after chukkas. Results indicated that number two ponies spent more time in zone 4 ($p < 0.05$) but less in zone 2 ($p < 0.01$) than the number four ponies. Cortisol levels increased immediately and 30 minutes afterward ($p < 0.0001$ for both periods) and then returned to baseline 60–90 minutes after exertion. Other measures (Hct, Hb, RBC, WBC, neutrophils, and CK enzyme) increased immediately ($p < 0.0001$ for all variables) and lasted at least 180 minutes after exertion ($p < 0.05$–0.0001). HRV decreased during the chukka until approximately 90 minutes afterward ($p < 0.05$–0.0001). The stress index increased during the chukka and declined to baseline at 60 minutes in number 1–3 ponies but lasted 90 minutes in those at number four. Effort intensity distribution differed among field-play positions. Decreased HRV indicated reduced parasympathetic activity, corresponding to increased sympathetic activity during warm-up and workout, extending to 90 minutes after exertion. Defenders seem to experience more stress than those in other positions.

Keywords

effort intensity; polo; field-play position; horse; physiology; stress

Introduction

Horses are capable of high-intensity exercise because they can increase oxygen consumption, extraction, and cardiac output during explosive efforts such as escaping from predators [1]. However, horses utilized for sports may face appalling conditions involving exogenous and endogenous stressors during their career [1]. Stress following involuntary activities in equestrian sports could lead to reduced performance, homeostasis disturbance, and medical problems in the body systems involved in those efforts [2, 3].

Unlike other equestrian sports, polo is the only one where team pairs play against one another [4]. Each team comprises four combinations in which the player's abilities are classified from –2 (low performance) to +10 (high performance). The total handicap of a team's players is summed to account for the team's handicap (goals) [4, 5]. Played on about a 10-acre (4.05 ha) grass field, a game consists of four, five, and six chukkas in low, medium, and high-goal competitions [6]. Polo horses experience rapid speed acceleration, pauses, and swift direction changes throughout the 7 minutes of each chukka [4, 5, 7]. This could lead to impaired homeostasis and potentially compromise horse welfare during the match, especially in unfit horses.

Increased red [4, 5] and white blood cell [5] parameters have been observed immediately after polo matches. Furthermore, creatine kinase (CK) enzyme and lactate levels are also modulated [4, 8, 9], and blood pH and total carbon dioxide decrease, coinciding with increased base excess and lactate levels; these changes reflect that exertion during polo matches is at high-intensity [4, 8]. Measured heart rate (in terms of percentage of maximum heart rate, $\%HR_{max}$) is frequently used to determine the cardiovascular demand and exercise intensity in sport horses in different disciplines [10, 11], including polo ponies [7, 12]. One study showed that, during a low-goal polo match, ponies spent most of the time at less than 90% HR_{max} but

a few minutes above 90%, suggesting that exercise during the match created moderate to high stress on the cardiovascular system [7].

Changes in various parameters can be studied to determine horses' stress responses, including cortisol levels [13–15], behavior [16–18], and heart rate variability (HRV) [19–22]. It is well-accepted that levels of cortisol, a glucocorticoid hormone generated by the adrenal cortex, are frequently used to monitor stress levels in multiple conditions, such as road transportation [14, 23], stress-related disease, [24] and during equestrian competition [13, 25, 26]. It has been reported that horses participating in eventing experience higher cortisol levels than those in jumping and dressage [15]. In contrast, endurance horses have the highest cortisol levels post-exercise, compared to the cross-country phase of 3-day events, trotting and galloping races, and jumping [27]. Hence, stress levels differ among equestrian sporting disciplines according to exercise intensity and the demands of competing in specific sports [2, 13, 27].

As an alternative to cortisol, HRV has also been used as a non-invasive marker of autonomic and physiological responses that can reflect stress levels and welfare [13, 14, 22, 28, 29]. HRV is a continuous measure of change in time differences between consecutive heartbeats during the cardiac cycle. Fluctuations in the interval between heartbeats are influenced by the interplay between sympathetic and parasympathetic (vagal) components that act on the sinoatrial node of the heart in conjunction with respiration and neurohormonal factors released into the blood circulation [30–33]. The irregular time interval between sequential heartbeats benefits the adaptability and flexibility of the body to cope with difficult situations [29, 33]. HRV appears to be a reliable indicator of autonomic responses in horses in several conditions, including specific veterinary protocols [34, 35], transportation [14, 36, 37], exercise training [20, 22, 38], and exercise during equestrian events [13, 39, 40]. Several HRV measures indicate the sympathetic and parasympathetic (vagal) activities in response to external stimuli.

HRV time domains, including verified beat-to-beat (RR) interval and standard deviation of normal-to-normal RR intervals (SDNN), reflect the long-term heart rate variation affected by sympathetic and vagal activities [29, 31]. The root mean square of successive RR interval differences (RMSSD), the number of consecutive RR interval pairs that differ by more than 50 msec (NN50), and its relative value (pNN50) all point to the short-term heart rate variation under the influence of vagal activity [33]. Triangular interpolation of normal-to-normal intervals (TINN) and the RR triangular index are geometric methods that express overall HRV during a measurement period [31, 41].

HRV frequency domains are also computed to indicate sympathetic and vagal activities. Modulation in the high-frequency (HF) band is affected mainly by vagal action, while the low-frequency (LF) band changes under sympathetic and vagal components [29, 31, 33, 42]. However, change in the very-low-frequency (VLF) band occurs following vagal activity, thermoregulation, renin-angiotensin, and vasomotor tone [31, 43, 44]. Nonlinear results, consisting of the standard deviation of the Poincaré plot perpendicular to the line of identity (SD1) and the standard deviation of the Poincaré plot along the line of identity (SD2), reflect short-term and long-term HRV, respectively [29, 33]. Furthermore, SD1 and RMSSD have been reported to be identical in reflecting short-term HRV and vagal activity [45], while SD2/SD1 ratio indicates sympathovagal balance and correlates with LF/HF ratio [31, 46].

Decreased RR interval, RMSSD, and SD1 reflect reduced vagal activity during short and medium-distance road transportation in horses [47]. On the contrary, increased RMSSD following aromatherapy indicated a shift toward vagal action [19]. Concerning equestrian competition, differences in RR interval, RMSSD, pNN50, SD1, and %VLF values were observed in horses jumping different fence heights [40]. A reduction in RMSSD was also documented in horses participating in equestrian sports [13, 21, 48]. In addition, the vast majority of HRV variables, including time domains (RR interval, SDNN, RMSSD, NN50,

pNN50, TINN, and RR triangular index), frequency domains (VLF, LF, and HF bands) and nonlinear results (SD1 and SD2) decreased in horses during exercise [22, 38]. Hence, HRV is a helpful indicator for determining autonomic responses in horses.

Since the exercise pattern of equestrian polo [4] is similar to team sports such as soccer [49], knowledge of physical and physiological responses may be transferred from human team sports to monitoring bodily responses in polo ponies. It has been reported that wingbacks perform more maximum exercise intensity than forwards during competitive soccer matches [50]. Moreover, from a young age, outfielders (forwards, midfielders, and defenders) displayed higher VO_2max, mean aerobic power, and sprint performance than goalkeepers [51]. Hence, different player positions experience distinct exercise intensities and physiological demands during soccer games.

Although there were reports on hematological and physiological responses in polo ponies participating in polo matches, responses in ponies across different field-play positions are scarce. Furthermore, the autonomic regulation of such ponies, particularly regarding their different positions during a match, has not yet been elucidated. Therefore, this study aimed to investigate the impact of traditional exercise regimens in polo on effort intensity distribution, cortisol levels, hematology, and autonomic regulation in ponies with different field-play positions.

Methods

Horses

We studied 32 healthy Argentine polo ponies (nine geldings and 23 mares, aged 12.46 ± 2.38 years, weighing 413.20 ± 10.37 kg) from the Thai Polo and Equestrian Club, Pattaya, Thailand. They had been regularly trained according to the traditional polo training method [12] and

playing two to four chukkas weekly during polo tournaments. They were housed separately in a 4 x 5 m stable within barns accommodating up to 40 horses. Daily feeding consisted of 2 kg of commercial pellets (provided across two meals) and 15 kg of hay hung permanently. Ponies had free access to tap water in their stables. They also spent approximately two hours daily in a paddock, except on match days. No ponies were undergoing medical or surgical treatment before the experiment commenced. The study protocol was approved by the Kasetsart University's Institute of Animal Care and Use Committee (ACKU65-VET-003).

Experimental protocol

The study was conducted in January 2022 during the Thai Polo and Equestrian Club tournament in Pattaya, Thailand. Thirty-two polo ponies were divided into eight teams, in which four player positions were deployed in a team, including number one (forwarder), numbers two and three (midfielders), and number four (defender). A team pair played against each other in four low-goal field plays outlined by the basic rules of polo [4]. The player's handicaps within the teams were 8.88 ± 2.42 goals. Since there were four chukkas, with a period of six and a half minutes per chukka, the ponies were assigned to participate only in the first chukka of each match for four consecutive days. The matches occurred in the late afternoon (15.00–17.00 h local time), during fine weather with an average humidity of 37.75 ± 2.75% and mean temperature of 35.60 ± 0.57 ºC. The relevant sampling was conducted before, during, and after the first chukka.

Hormonal and hematological analyses

Hormonal and hematological variables were determined before the chukka, immediately afterward, and at 30-minute intervals for a further 180 minutes. In brief, 9 ml of whole blood was withdrawn from the jugular vein and divided into two parts. Three ml of blood was placed in potassium EDTA tubes for evaluating hematocrit (Hct), red blood cells (RBC), hemoglobin concentration (Hgb), white blood cells (WBC), neutrophils, and lymphocytes using an

automated hematology analyzer (Advia®2120i; Siemens Healthineers, Erlangen, Germany). Another portion of the blood sample was placed in 6 ml clotting activator tubes to obtain serum for biochemical examination, including 1) cortisol level determination using a competitive chemiluminescent enzyme immunoassay (IMMULITE Analyzers, Siemens Healthineers, Erlangen, Germany) and expressed as nmol/L, and 2) evaluation of creatine kinase (CK) level using a Liquid NAC activated UV test (HUMAN Gesellschaft für Biochemica und Diagnostica mbH, Wiesbaden, Germany) and expressed as ukat/L.

Heart rate and heart rate variability analyses

Data collection

Ponies were equipped with portable Polar heart rate monitoring (HRM) device sets (Polar Electro Oy, Kempele, Finland) for HR and HRV analyses. The device set comprises a Polar heart rate equine belt for trotters, a heart rate sensor (Polar H10), and a sports watch for recording RR intervals (Polar vantage 2). In brief, the heart rate sensor was attached to the equine belt for trotters at the sensor pocket on the belt and then soaked in water to increase signal transmission. The soaked belt was fastened around the pony's chest, where the sensor was placed on the left side of the chest. The sensor was connected to the sports watch to record RR interval data 15 minutes before the chukka. The players wore sports watches for recording RR intervals during the competition, which were then left close to the ponies with whom the sensors were paired for 180 minutes after the competition.

Data acquisition

RR interval data from the sports watch was transferred to the Polar Flow program (https://flow.polar.com/) for HRV analysis. HRV variables were later computed from RR interval data using Kubios Premium software (Kubios HRV Scientific; https://www.kubios.com/hrv-premium/) and exported as the MATLAB MAT-file (S1 fig.). The premium software provides the automatic correction algorithm, which has been validated

to correct artifacts and ectopic beats in the interbeat interval (IBI) data; in turn, this produces more accurate HRV variables than the standard version [52]. The Kubios HRV Scientific software also supports automatic noise detection to identify noise segments that distort various consecutive beat detections in the IBI data. The automatic noise detection was set at a medium level in this study. Before analyzing HRV variables, smoothness priors were used to remove IBI time series nonstationarities. According to the user guideline manual, the cutoff frequency was fixed at 0.035 Hz (https://www.kubios.com/downloads/Kubios_HRV).

The HRV variables were as follows:

Time domain results: RR interval, HR, SDNN, RMSSD, pNN50, TINN, RR triangular index, stress index, heart rate deceleration capacity computed as a four-point difference (DC) index, and modified DC computed as a two-point difference (DCmod).

Frequency domain results: VLF band (by default 0–0.04 Hz), LF band (by default 0.04–0.15 Hz), HF band (by default 0.15–0.4 Hz), LF/HF ratio, total power spectrum, VLF/total power ratio, LF/total power ratio, HF/total power ratio and respiratory rate (RESP).

Nonlinear results: SD1 and SD2.

Autonomic nervous system (ANS) indexes: SD1% ([SD1 / (SD1 + SD2)] × 100), SD2% ([SD2 / (SD1 + SD2)] × 100), parasympathetic nervous system (PNS) index and sympathetic nervous system (SNS) index.

The HRV variables were determined 15 minutes before the match began, during the six and a half minutes of the first chukka, and at 30-minute intervals for 180 minutes after it.

Time spent within effort intensity zones

Real-time HR modulations during each chukka were derived from the Polar HRM device. The ponies' effort intensity during the chukka was estimated using the percent maximum HR (%HR_{max}), similar to previous reports in a competitive soccer match [50]. The %HR_{max} was

calculated using horses' reference maximum heart rate at 220 beats/min [(recorded HR x100)/220], according to previous work [12]. The computed $\%HR_{max}$ of the ponies was then categorized into five zones, including zone 1 (<70% HR_{max}), zone 2 (70–80% HR_{max}), zone 3 (80–90% HR_{max}), zone 4 (90–95% HR_{max}), and zone 5 (>95% HR_{max}). The distributions of time spent within each zone were determined in each pony.

Speed and distance covered

The ponies' riding speed and distance covered were recorded by the Polar sports watches worn on the players' wrists throughout the chukka and exported as km/h and kilometers, respectively.

Statistical analysis

HR and HRV data were analyzed using GraphPad Prism version 10.1.0 (GraphPad Software Inc, San Diego, USA). In an analysis of two-grouping variables, two-way ANOVA with repeated measures was applied to evaluate the independent effects of field-play position and time, as well as the interaction effect of field-play position and time, on changes in cortisol level, hematological, HR, and HRV variables in response to the exertion during competition. Dunnett's *post-hoc* test was later implemented to assess between-group differences at given time points and within-group differences compared to the control (baseline values taken before competition). Tukey's *post-hoc* test was also applied when necessary. Two-way ANOVA was further used to determine the independent effects of field-play position and effort intensity zone ($\%HR_{max}$) and their interaction on the time-spent distribution within zones. Tukey's *post-hoc* test was implemented to compare time spent in different effort zones within and between field-play positions.

Regarding the one-grouping variable analysis, the data's normal distribution was verified by the Shapiro-Wilk test. Due to the data being normally distributed, an ordinary one-

way ANOVA followed by Tukey's *post-hoc* test was employed to estimate differences in riding speed and covered distance among the ponies playing in different positions. The Kruskal-Wallis test, accompanied by Dunn's multiple comparisons tests, was alternatively applied to assess players' handicaps among different field-play positions because the data showed a non-normal distribution. Data were expressed as mean ± SD, and $p < 0.05$ was considered statistically significant.

Results

Player's handicap, riding speed, and distance covered

The average handicap of the number four players was higher than number one players (3.75 ± 1.39 vs. 1.38 ± 1.06, $p = 0.0170$) (Fig. 1a). The average riding speed and covered distance did not differ among the field play positions (Figs. 1b and c).

Fig. 1. Player handicap (a), average riding speed (b), and covered distance of polo ponies during field-play competition. *indicates statistical significance between pairs of comparison at $p < 0.05$. **P-1**; horse played at number 1 position, **P-2**; horse played at number 2 position, **P-3**; horse played at number 3 position, and **P-4**; horse played at number 4 position.

Time spent within different effort zones

There was an interaction between field-play position and effort intensity zone (%HR_{max}) ($p = 0.0497$) and the separate effect of effort intensity zone ($p < 0.0001$) on the distribution of time spent within the zones. The ponies at the number one position spent more time in zone 3 compared to zones 1 ($p < 0.05$), 4 ($p < 0.05$) and 5 ($p < 0.01$). The ponies at number two position spent more time in zone 3 than in zones 2 and 5 ($p < 0.05$ for both), and in zone 4 more than zone 5 ($p < 0.01$). The time spent among different zones by number three ponies did not vary. The number four ponies spent more time in zones 2 and 3 than both zone 4 ($p < 0.05$ for both)

and zone 5 ($p < 0.001$ for both). The number four ponies spent more time in zone 2 ($p < 0.01$), and less time in zone 4 ($p < 0.01$), compared with number two ponies (Fig. 2).

Fig. 2. Percentage time spent in each heart rate (HR) zone in polo ponies at different field-play positions 1-4 (P-1, P-2, P-3 and P-4). *indicates interaction *p*-value of analysis. Different letters (a, b, and c) indicate the statistical significance among HR zones within particular field-play positions. *indicates significant differences in HR zones between positions. Zone 1 (<70% HRmax: HR<154 bpm), Zone 2 (70–80% HRmax:155≤HR≤176 bpm), Zone 3 (80–90% HRmax: 177≤HR≤198 bpm), Zone 4 (90–95% HRmax: 199≤HR≤209 bpm) and Zone 5 (>95% HRmax: HR>210 bpm).

Hormonal and hematological analyses

Since there was no interaction effect between group and time on changes in hormonal levels and hematological parameters, the parameters were subsequently evaluated as a pool, following the main effect of time. Cortisol levels increased immediately after the match and remained high until 30 minutes afterward ($p < 0.0001$ for both periods). After reducing to the baseline value at 60–90 minutes after the match, cortisol levels decreased to below the baseline at 120–180 minutes ($p < 0.0001$ for all periods) (Table 1).

CK levels increased immediately until 150 minutes after the match ($p < 0.05$–0.0001). At the same time, hematological parameters (including Hct, Hb, and RBC) increased immediately until 180 minutes afterward ($p < 0.0001$ for given periods of all variables). Even though WBC and neutrophil levels rose immediately until 180 minutes after competition ($p < 0.0001$, except for $p < 0.05$ at 60 minutes after competition for both variables), lymphocytes were unchanged throughout the study period (Table 1).

Table 1. Biochemical and hematological parameters (mean ± SD) in polo ponies (n=32) before and after low-goal polo competition.

Parameters	Before competition	After competition						
		Immediately	30 min	60 min	90 min	120 min	150 min	180 min
**Cortisol** (25–155 nmol/L)	68.89± 10.19	115.53± 6.44[d]	101.91± 4.7[d]	75.70± 6.67	58.89± 6.12	45.14± 4.44[d]	39.61± 0.85[d]	31.67± 0.5[d]
**Creatine kinase (CK)** (0.17-5.83 ukat/L)	4.06± 0.24	4.64± 0.36[d]	4.70± 0.49[b]	5.18± 0.63[b]	5.87± 0.83[b]	5.57± 0.67[b]	5.48± 0.78[a]	5.27± 0.83
**Hematocrit (Hct)** (32-53%)	32.29± 0.73	49.02± 1.04[d]	37.61± 1.11[d]	36.11± 1.77[d]	37.82± 0.66[d]	38.61± 1.12[d]	37.79± 1.04[d]	37.26± 0.5[d]
**Hemoglobin (Hb)** (110-190 g/L)	117.28± 2.18	171.66± 3.28 [d]	133.94± 3.4[d]	128.56± 3.4[d]	133.88± 1.7[d]	136.34± 3.0[d]	134.28± 2.8[d]	132.63± 1.60[d]
**Red blood cell (RBC)** (6.80-12.90 x 10^{12}/L)	6.89± 0.11	10.25± 0.31[d]	7.96± 0.25[d]	7.63± 0.21[d]	7.97± 0.21[d]	8.14± 0.25[d]	7.96± 0.30[d]	7.86± 0.25[d]
**White blood cell (WBC)** (5.40-14.30 x 10^{9}/L)	6.48± 0.12	8.47± 0.31[d]	7.23± 0.16[d]	6.87± 0.35[a]	7.61± 0.11[d]	7.79± 0.27[d]	7.80± 0.27[d]	7.84± 0.28[d]
**Neutrophil (Neu)** (2.26-8.50 x 10^{9}/L)	4.30± 0.39	5.72± 0.39[d]	4.80± 0.30[a]	4.89± 0.54[a]	5.50± 0.36[d]	5.66± 0.05[d]	5.69± 0.24[d]	5.62± 0.24[d]
**Lymphocyte (Lymp)** (1.50-7.70 x 10^{9}/L)	2.02± 0.31	2.49± 0.25	2.23± 0.40	1.75± 0.25	1.88± 0.36	1.86± 0.21	1.79± 0.23	1.95± 0.17

a, **b** and **d** indicate statistical differences of given time points compared to before competition values at $p < 0.05$, $p < 0.01$, and $p < 0.0001$, respectively.

Heart rate and heart rate variability

Time domain results

Only time exerted effects on HR and most time domain HRV variables, except for stress index, which demonstrated a group-by-time interaction ($p = 0.0322$), in addition to the independent effect of time.

The minimum HR increased during the warm-up and competition period ($p < 0.0001$ for both periods). Despite a gradual reduction, the minimum HR remained higher than the baseline values until 180 minutes after the competition ($p < 0.0001$ for all periods) (Fig. 3a). The maximum HR also rose dramatically during the warm-up and peaked during the competition ($p < 0.0001$ for both periods). It was still high 30 minutes after the competition ($p < 0.0001$) and then reduced considerably to the baseline 60–90 minutes afterward. Maximum HR decreased further, to below the baseline, at 120 ($p < 0.05$), 150 ($p = 0.0728$), and 180 minutes ($p < 0.05$) after the match (Fig 3b).

As expected, mean HR increased during the warm-up and competition ($p < 0.0001$ for both periods). After that, it reduced dramatically 30 minutes after the competition but remained higher than the baseline ($p < 0.0001$). Although it continued to decrease, it remained higher than the baseline 180 minutes after the match ($p < 0.05$) (Fig. 3c).

Fig. 3. Modification in minimum HR (a), maximum HR (b), mean HR (c), and RR intervals (d) in response to exercise in polo matches. *indicates the interaction *p*-value of all variables. **a, b, c,** and **d** letters indicate the statistical significance of each time point compared to the control at $p < 0.05$, $p < 0.01$, $p < 0.001$, and $p < 0.0001$, respectively. **P-1**; horse played at number 1 position, **P-2**; horse played at number 2 position, **P-3**; horse played at number 3 position, and **P-4**; horse played at number 4 position.

In contrast to HR variables, the mean RR interval decreased during warm-up ($p < 0.00001$) and plunged to its lowest value during competition ($p < 0.0001$). There was a sharp rise in mean RR intervals 30 minutes ($p < 0.0001$) after the match. However, they then reached a plateau and remained higher than the baseline 60–180 minutes after the competition ($p <$ 0.01–0.001) (Fig. 3d). SDNN, RMSSD, and pNN50 shared the same trend: they dropped dramatically during warm-up ($p < 0.0001$ for all variables), then continued to decrease to their lowest values during the competition ($p < 0.0001$ for all variables). After competition, they increased markedly 30–90 minutes ($p <$ 0.001–0.0001 for all variables) until reaching the baseline at 120 minutes. In addition, the pNN50 was higher than the baseline at 180 minutes after the match ($p < 0.05$) (Figs. 4a–c).

Since an interaction was observed between field-play position and time, the stress index differed among the ponies in the match. The stress index increased during warm-up (number one ponies, $p < 0.01$; number two, $p < 0.05$; number three, $p < 0.001$; number four, $p < 0.01$) and peaked during competition periods ($p < 0.0001$ for number one, two and three ponies and $p < 0.001$ for number four ponies). It then reduced sharply 30 minutes after competition

(number one ponies, $p < 0.001$; number two, $p < 0.05$; number three, $p < 0.01$; number four, $p < 0.001$). Although the stress index decreased to the baseline 60 minutes after competition in the ponies at numbers 1–3, it later reduced to the baseline 120 minutes after competition in those at number four ($p < 0.01$) (Fig. 4d).

Fig. 4. Time domain analysis of HRV in response to exercise in polo matches, including standard deviation of RR intervals (SDNN) (a), square root of the mean squared differences between successive RR intervals (RMSSD) (b), relative number of successive RR interval pairs that differ by more than 50 msec (pNN50) (c), and stress index (d). *indicates the interaction *p*-value of all variables. **a, b, c,** and **d** letters indicate the statistical significance of each time point compared to the control at $p < 0.05$, $p < 0.01$, $p < 0.001$, and $p < 0.0001$, respectively. **α**, **β**, **γ**, and **ε** indicate significant differences in each time point compared to their controls in P-1, P-2, P-3 and P-4, respectively. **P-1**; horse played at number 1 position, **P-2**; horse played at number 2 position, **P-3**; horse played at number 3 position, and **P-4**; horse played at number 4 position.

TINN and RR triangular index decreased during warm-up ($p < 0.0001$ for both variables) and were lowest during the competition ($p < 0.0001$ for both) (Figs. 5a and b). TINN increased 30 minutes after the match ($p < 0.0001$) and returned to the baseline 60 minutes later (Fig. 5a). In contrast, the RR triangular index was higher at 30–90 minutes ($p <$ 0.05–0.0001) and reached the baseline 120 minutes after the match. However, the RR triangular index rose further and was higher than the baseline 150–180 minutes afterward ($p <$ 0.01–0.001) (Fig. 5b). DC and DCmod reduced during warm-up, with the lowest values during the match ($p < 0.0001$ in both periods of the two variables). They increased 30–90 minutes after the match (DC, $p <$ 0.01–0.0001; DCmod, $p <$ 0.001–0.0001) and reached the baseline 120 minutes later (Figs. 5c and d).

Fig. 5. Modulation in time domain analysis, including triangular interpolation of normal-to-normal intervals (TINN) (a), RR triangular index (b), heart rate deceleration capacity computed as a four-point difference (DC) (c), and modified DC computed as a two-point difference (Dcmod) (d) in response to exercise in polo matches. *indicates the interaction *p*-value of all variables. **a, b, c**, and **d** letters indicate the statistical significance of each time point compared to the control at $p < 0.05$, $p < 0.01$, $p < 0.001$, and $p < 0.0001$, respectively. **P-1**; horse played at number 1 position, **P-2**; horse played at number 2 position, **P-3**; horse played at number 3 position, and **P-4**; horse played at number 4 position.

Frequency domain results

VLF, LF, and HF bands decreased during warm-up (VLF and LF, $p < 0.0001$; HF, $p < 0.01$) and the competition ($p < 0.0001$ for all variables) (Figs. 6a–c). VLF band rose 30–120 minutes ($p < 0.05$–0.0001) and returned to the baseline 150 minutes later (Fig. 6a). LF band increased from 30 ($p < 0.0001$) to 60 minutes ($p < 0.01$) and reached the baseline 90 minutes later (Fig 6b). In the meantime, the HF band returned to the baseline earliest, at 60 minutes after the match ($p < 0.001$) (Fig. 6c). LF/HF ratio increased during the match ($p < 0.0001$) and returned to the baseline 30 minutes later (Fig. 6d).

Fig. 6. Changes in frequency domain analysis, including very-low-frequency band (VLF) (a), low-frequency band (LF) (b), high-frequency band (HF) (c), and LF/HF ratio (d) in response to exercise in polo matches. *indicates the interaction *p*-value of all variables. **a, b, c**, and **d** letters indicate the statistical significance of each time point compared to the control at $p < 0.05$, $p < 0.01$, $p < 0.001$, and $p < 0.0001$, respectively. **P-1**; horse played at number 1 position, **P-2**; horse played at number 2 position, **P-3**; horse played at number 3 position, and **P-4**; horse played at number 4 position.

The total power spectrum reduced from the warm-up period ($p < 0.01$), with the lowest value during the match ($p < 0.0001$), extending to 90 minutes afterward ($p < 0.05$–0.0001) (Fig.

7a). The VLF/total power ratio increased during the match ($p < 0.05$), then reduced sharply 30 minutes ($p < 0.0001$) and 120–150 minutes afterward ($p < 0.01$–0.001) (Fig. 7b). LF/total power ratio was unchanged throughout the study period (Fig. 7c), while HF/total power ratio decreased during the match ($p < 0.0001$) (Fig. 7d). RESP rose from the warm-up period to 30 minutes after the match ($p < 0.0001$ for all periods). However, it declined to the baseline during 60–90 minutes and then rebounded above the baseline 120–180 minutes afterward ($p < 0.05$–0.01) (Fig. 8a).

Fig. 7. Total power spectrum (a), VLF/total power ratio (b), LF/total power ratio (c), and HF/total power ratio (d) in response to exercise in polo matches. *indicates the interaction *p*-value of all variables. **a, b, c**, and **d** letters indicate the statistical significance of each time point compared to the control at $p < 0.05$, $p < 0.01$, $p < 0.001$, and $p < 0.0001$, respectively. **P-1**; horse played at number 1 position, **P-2**; horse played at number 2 position, **P-3**; horse played at number 3 position, and **P-4**; horse played at number 4 position.

Nonlinear results

SD1 and SD2 also decreased during warm-up and the match ($p < 0.0001$ for both periods of the two variables). They increased 30–90 minutes after the match ($p < 0.001$–0.0001 for both periods of the two variables) and reached the baseline 120 minutes afterward (Figs. 8b and c). An increase in SD2/SD1 was observed during the warm-up and competition ($p < 0.0001$ for both periods) (Fig. 8d).

Fig. 8. Changes in respiratory rate (RESP) (a), standard deviation of the Poincaré plot perpendicular to the line of identity (SD1) (b), standard deviation of the Poincaré plot along the line of identity (SD2) (c), and SD2/SD1 ratio (d) in response to exercise in polo matches. *indicates the interaction *p*-value of all variables. **a, b, c**, and **d** letters indicate the statistical significance of each time point compared to the control at $p < 0.05$, $p < 0.01$, $p < 0.001$, and $p<0.0001$, respectively. **P-1**; horse played at number 1 position, **P-2**; horse played at number

2 position, **P-3**; horse played at number 3 position, and **P-4**; horse played at number 4 position.

ANS indexes

SD1% decreased, corresponding to an increase in SD2% during warm-up and competition ($p < 0.0001$ for both periods) (Figs. 9a and b). The PNS index declined after the warm-up and plunged to its lowest value during the match ($p < 0.0001$ for both periods). It then rose 30–120 minutes after it ($p < 0.05$–0.0001) and returned to the baseline by 150 minutes afterward (Fig. 9c). The SNS index increased during the warm-up and peaked during the match ($p < 0.0001$ for both periods). A sharp decrease was detected 30 minutes after ($p < 0.0001$), then gradually reduced between 60–90 minutes after the match ($p < 0.001$–0.0001) (Fig 9d).

Fig. 9. Autonomic nervous system index, including SD1% (a), SD2% (b), parasympathetic nervous system (PAS) index (c), and sympathetic nervous system (SAS) index (d) in response to exercise in polo matches. *indicates the interaction *p*-value of all variables. **a, b, c**, and **d** letters indicate the statistical significance of each time point compared to the control at $p < 0.05$, $p < 0.01$, $p<0.001$, and $p < 0.0001$, respectively. **P-1**; horse played at number 1 position, **P-2**; horse played at number 2 position, **P-3**; horse played at number 3 position, and **P-4**; horse played at number 4 position.

Discussion

The present study determined the impact of a classic exercise regimen in a polo match on effort distribution, cortisol release, hematology, and heart rate variability. The significant findings from this study were: 1) number two ponies spent more time exercising in effort intensity zone 4, but less in zone 2, than number four ponies. 2) There were no differences in cortisol levels and hematological modulation among ponies with different field-play positions. 3) Cortisol levels rose considerably during competition and returned to the baseline 60 minutes later. 4)

Hct, RBC, Hb, WBC, neutrophils, and CK levels increased immediately and remained higher than the baseline up to 180 minutes after exertion. 5) Mean HR increased considerably and remained higher than the baseline at 180 minutes. 6) HRV modulation did not differ among ponies in different field-play positions, except for the stress index, which declined later in the number four ponies than those in other field-play positions. 7) HRV decreased to the lowest values during exercise, gradually increasing and returning to baseline 120 minutes afterward.

The results suggest that polo ponies at different field-play positions experienced different levels of effort intensity. A decreased HRV reflected a shift toward sympathetic dominance during warm-up, with a substantial sympathetic effect during the workout, which lasted for approximately 90 minutes afterward. The number four ponies appeared to experience more stress than those in other positions, as indicated by the stress index modification. To our knowledge, this is the first study to report the physiological demands and autonomic responses in polo ponies with different field-play positions in low-goal polo matches.

In soccer, players' physical and physiological demands also vary across different positions [50, 51, 53]. Wingbacks spend more time in higher-intensity zones than forwards [50]. Wingbacks typically must participate in offensive action along with the forwards, alternating with a sprint back to cover opponents in a defensive system. In contrast, forwards and defenders spent more time with lower exercise intensity than other player positions because their specific task was scoring [50]. It is worth noting that specific training regimens, with appropriately adjusted workloads, can increase players' performance in different positions. Since equestrian polo and soccer have similar exercise patterns, differences in the positional demands are also expected in this equestrian sport.

In polo, each field-play position serves a distinct role. The number one position is usually filled by a player with relatively low experience, who is mainly responsible for scoring and neutralizing the opposite number four. In contrast, the number two player acts as a

scrambler who needs a keen eye and fast ponies to scrap for the ball. They may feed the ball to the number one player or score alone. The number three player is the primary tactical player who provides the ball to the number one and two players, while the number four plays a crucial role in the defensive system to prevent the opposition from scoring. In this study, the number two ponies spent more time in the higher effort zone and less time in the lower effort zones than those at number four. This seems consistent with the multifunctional role of the number two player [6]. In contrast, spending more time in lower effort zones in number four ponies may be consistent with their primary role in defense, undergoing multiple changes in direction and speed to prevent scoring; consequently, the running speed of number four ponies may not reach the high values observed in number two ponies. Our data thus suggest that ponies' physical effort within the chukka varies among different field-play positions and that specific training may be required for each position to reach the required performance at the respective intensity levels.

Serum cortisol levels and hematological parameters changed during the matches, regardless of field-play positions. Cortisol levels are known to be modulated in horses participating in various equestrian sports [13, 26, 27], including polo [5, 54]. The modulation we observed was consistent with previous studies in horses [55, 56] and polo ponies specifically [5], showing a sharp increase in serum cortisol immediately, lasting 30 minutes, and returning to baseline 60 minutes after exertion. These results reflect the enormous stress on the ponies during high-intensity exercise in the chukkas. However, a further decrease in cortisol levels (below the baseline) at 120–180 minutes may result from the hormone's circadian rhythm during the late evening [22, 57–60].

The observed increase in CK enzyme levels parallels those of a previous report [5], demonstrating a rise in after the chukkas, but within the normal reference range of the enzyme. Increased CK levels are believed to accompany high-intensity exercise due to muscle

microtrauma, leading to increased fiber membrane permeability, as reported elsewhere [49, 61]. Increases in Hct, Hb, RBC, and WBC after exertion were also observed, corresponding to previous studies [4, 5]. It is well-established that red blood cell parameters rise following splenic contraction to increase oxygen-carrying capacity during intense exercise [4, 5, 62, 63]. Furthermore, increased WBC levels are thought to be due to a shift into the bloodstream of lymphocyte [64] and neutrophil subpopulations [65] from the spleen, bone marrow, and lymph nodes. These reactions are influenced by cortisol and catecholamine releases following physical effort [66, 67].

Although the enhancement of WBC levels immediately after chukkas was similar to changes reported by Zobba et al. [5], a disparity in leucocyte modulation and distribution was detected compared to their study. They demonstrated that a transient rise in WBC was observed immediately after the chukka [5], compared to a more prolonged increased WBC count immediately and until 180 minutes after the chukka in our study. Moreover, the presence of increased lymphocytes, but not neutrophils, in polo ponies of the previous study [5] contrasted with our findings, which showed increased neutrophils instead of lymphocytes. Since a change in leukocyte distribution and magnitude of leucocytosis are related to exercise intensity and duration [68, 69], a difference in effort intensities (indicated by HR during the match) between the previous (~80 beats/min) and current (~175 beats/min) studies may be the underlying reason for the discrepancy. Although the ponies in this study showed increased serum cortisol levels and hematological profile after the chukkas, all parameter changes were within the normal reference range [5, 70]. These laboratory results may reflect the ponies' capability for physiological adjustment during physical effort in polo matches.

Concerning autonomic regulation during exertion, HRV typically decreases during conventional exercise in distinct equestrian sports [13, 39, 40, 71]. A lower HRV indicates a synchronous increase in sympathetic tone and decreased vagal tone or independent action of

increased sympathetic or reduced vagal activities [29, 33]. It has been reported that RMSSD decreased during competitive exercise but did not differ between horses in jumping and dressage events [13]. However, a difference in autonomic responses to ultra-short-term stimuli was observed in sports horses. Villas-Boas et al. (2022) demonstrated a lower LF/HF ratio—indicating a minimal autonomic response to the startle test—in endurance horses compared with dressage and polo horses [54]. It was suggested that autonomic response to startling challenges, but not exercise, differed among horses in different fields. In this study, a decrease in HRV variables derived by time domain (SDNN, RMSSD, pNN50, TINN, RR triangular index, DC, and DCmod), frequency domain (VLF, LF, and HF bands), and nonlinear (SD1 and SD2) methods was detected, regardless of field play positions, during exercise in the warm-up and within the chukka. These reductions in HRV were consistent with previous reports describing HRV modulation in response to exercise [22, 38], mirroring a decreased role of the vagal component and a shift toward sympathetic activity during exercise in polo. More importantly, higher HR indicated greater effort intensity during the chukka than during warm-up period. The increased effort intensity corresponded to a further decrease in HRV variables and, in turn, a progressive decrease in vagal activity during the chukka. These results indicated that exercise intensity was a contributing factor to HRV modification. Even though the frequency domain variables (VLF, LF, HF, and total power bands) decreased during the warm-up and the chukka, the contribution of each power spectrum band to the total power spectrum during the chukka differed from other given periods in the study.

LF/total power ratio remained unchanged throughout the study. In contrast, VLF/total power ratio increased, coinciding with a decreased HF/total power ratio during exercise. These results indicated a major contribution of the VLF band, corresponding to a decreased HF band, to the total power spectrum during exercise. Since the VLF band is modulated in response to vasomotor tone, thermoregulation, and renin-angiotensin action [72], an increased proportion

of VLF contribution may, at least in part, point out the apparent effect of vasomotor tone thermoregulation and renin-angiotensin action, along with sympathetic dominance, on autonomic regulation during the chukka. An increase in LF/HF ratio, SD2/SD1 ratios, SNS index, and SD2%, coinciding with reduced PNS index and SD1%, provided supporting evidence of an increased sympathetic and decreased vagal activity during exertion. Despite no differences in the HRV variable, the stress index differed among field-played positions. The stress index was reduced later among ponies in the defensive position than those playing in other positions, suggesting that defenders tend to be under more stress. This observation is partly consistent with a previous study reporting that defenders had the highest anaerobic power utilization, leading to reduced blood pH compared with those in other positions [8]. Accordingly, more care may be taken in selecting ponies for defensive positions to avoid compromised horse welfare in equestrian polo.

Although we report differences in hormonal, hematological, and autonomic responses according to the positions in which ponies compete in the first chukka of a low-goal match, the question arises as to what extent such modulations would occur in ponies competing in more than one chukka. In addition, the effect of age on the effort intensity distribution and HRV modification in such ponies needs further investigation. This study's main limitation was that various players rode the ponies without controlling for their weight. The potential impact of different loading on physiological responses in polo ponies during the games may be considered in the future.

Conclusion

The effort intensity distribution differed among polo ponies in different field-play positions during the chukka. Blood cortisol levels and hematological parameters did not vary with position. However, cortisol levels increased immediately and 30 minutes after the match. In contrast, red and white blood cell variables (except lymphocytes) increased immediately to 180

minutes after matches, while CK enzyme levels rose until 150 minutes afterward. A marked decrease in HRV indicated a shift toward sympathetic activity and physiological stress during warm-up and competition until 90 minutes afterward. The increased stress index of ponies at the number 4 position lasted longer than the other positions, implying that ponies in the defensive position tended to experience more stress than other positions. These results provide insight into effort intensity distribution, hematology, and autonomic regulation in response to exertion in polo ponies at different field-play positions. The information may, at least in part, be beneficial for selecting appropriate polo ponies regarding adequate fitness and proper autonomic function or adding specific training programs to reach appropriate fitness levels for typical field-play positions in polo, especially the number 2 and 4 ponies. More importantly, compared to before the game, the finding that expression of sympathetic dominance lasts 90 minutes after the match could heighten awareness of the prolonged physiological stress in polo ponies, despite playing only one chukka.

Declaration of competing interests

The authors declare that they have no competing interests.

Acknowledgments

We would like to thank Harald Link, the president of the Thailand Equestrian Federation (TEF), and Nunthinee Tanner, the vice president, for allowing us to conduct the experiment at the Thai Polo and Equestrian Club. Surapol Puthapitak and Manu Cereceda, the club's manager, are also thanked for their tremendous support in facilitating data collection.

Author contributions

Kanokpan Sanigavatee, Chanoknun Poochipakorn, Onjira Huangsaksri and **Thita Wonghanchao** substantially contributed to conceptualization, data curation, formal analysis,

investigation, methodology, software, validation, visualization, writing – original draft preparation.

Mona Yalong, Kanoknoot Poungpuk and Kemika Thanaudom contributed to data curation, formal analysis, investigation, validation and visualization, writing – original draft preparation.

Metha Chanda was responsible for conceptualization, data curation, formal analysis, funding acquisition, investigation, methodology, project administration, resources, software, supervision, validation, visualization, writing – original draft preparation and writing – review & editing. All authors approved the final version of the manuscript.

Data availability

The HRV data supporting this study's findings are available at https://doi.org/10.6084/m9.figshare.24785247.v1

References

1. Art T, Lekeux P. Ventilatory and arterial blood gas tension adjustments to strenuous exercise in standardbreds. American Journal of Veterinary Research. 1995; 56(10):1332–1337.
2. Bartolomé E, Cockram MS. Potential Effects of stress on the performance of sport horses. J Equine Vet Sci. 2016; 40:84–93.
3. Folsom RW, Littlefield-Chabaud MA, French DD, Pourciau SS, Mistric L, Horohov DW. Exercise alters the immune response to equine influenza virus and increases susceptibility to infection. Equine Vet J. 2001; 33(7):664–669.
4. Ferraz GC, Soares OAB, Foz NSB, Pereira MC, Queiroz-Neto A. The workload and plasma ion concentration in a training match session of high-goal (elite) polo ponies. Equine Vet J. 2010; 42(s38):191–195.
5. Zobba R, Ardu M, Niccolini S, Cubeddu F, Dimauro C, Bonelli P, et al. Physical, hematological, and biochemical responses to acute intense exercise in polo horses. J Equine Vet Sci. 2011; 31(9):542–548.
6. Meulemans P. The basic rules of polo. Museumplein Polo Amsterdam [Available from: https://www.museumpleinpoloamsterdam.com/en/basic-rules-of-polo/].
7. Marlin DJ, Allen JCR. Cardiovascular demands of competition on low-goal (non-elite) polo ponies. Equine Vet J. 1999; 31(5):378–382.
8. Gondin MR, Foz NSB, Pereira MC, Flagliari JJ, Orozco CAG, D'Angelis FHF, et al. Acute phase responses of different positions of high-goal (elite) polo ponies. J Equine Vet Sci. 2013; 33(11):956–961.
9. Noleto PG, Cubas JPC, Barbosa FC, Guimarães EC, Mundim AV. Biochemical profile of polo horses in training phase and those players of official competition. Comp Clin Pathol. 2016; 25(4):911–915.
10. Muñoz A, Riber C, Santisteban R, Rubio MD, et al. Cardiovascular and metabolic adaptations in horses competing in cross-country events. J Vet Med Sci. 1999; 61(1):13–20.
11. Williams J, Kenworthy K, Jones T, Marlin D, Tabor G. The role of heart rate monitoring to assess workload during maintenance interval training in National Hunt racehorses. J Vet Behav. 2019; 30:54–60.
12. Chanda M, Srikuea R, Cherdchutam W, Chairoungdua A, Piyachaturawat P. Modulating effects of exercise training regimen on skeletal muscle properties in female polo ponies. BMC Vet Res. 2016; 12(1):245.
13. Becker-Birck M, Schmidt A, Lasarzik J, Aurich J, Möstl E, Aurich C. Cortisol release and heart rate variability in sport horses participating in equestrian competitions. J Vet Behav. 2013; 8(2):87–94.
14. Schmidt A, Hödl S, Möstl E, Aurich J, Müller J, Aurich C. Cortisol release, heart rate, and heart rate variability in transport-naive horses during repeated road transport. Domest Anim Endocrinol. 2010; 39(3):205–213.
15. Strzelec K, Kankofer M, Pietrzak S. Cortisol concentration in the saliva of horses subjected to different kinds of exercise. Acta Vet Brno. 2011; 80(1):101–105.
16. Budzyńska M. Stress reactivity and coping in horse adaptation to environment. J Equine Vet Sci. 2014; 34(8):935–941.
17. Covalesky ME, Russoniello CR, Malinowski K. Effects of show-jumping performance stress on plasma cortisol and lactate concentrations and heart rate and behavior in horses. J Equine Vet Sci. 1992; 12(4):244–251.
18. Destrez A, Grimm P, Cézilly F, Julliand V. Changes of the hindgut microbiota due to high-starch diet can be associated with behavioral stress response in horses. Physiol Behav. 2015; 149:159–164.

19. Baldwin AL, Chea I. Effect of Aromatherapy on Equine Heart Rate Variability. J Equine Vet Sci. 2018; 68:46–50.
20. Cottin F, Médigue C, Lopes P, Petit E, Papelier Y, Billat VL. Effect of exercise intensity and repetition on heart rate variability during training in elite trotting horse. Int J Sports Med. 2005; 26(10):859–867.
21. Frippiat T, van Beckhoven C, van Gasselt VJ, Dugdale A, Vandeweerd JM. Effect of gait on, and repeatability of heart rate and heart rate variability measurements in exercising Warmblood dressage horses. Comp Exer Physiol. 2023 :1–12.
22. Poochipakorn C, Wonghanchao T, Huangsaksri O, Sanigavatee K, Joongpan W, Tongsangiam P, et al. Effect of exercise in a vector-protected arena for preventing african horse sickness transmission on physiological, biochemical, and behavioral variables of horses. J Equine Vet Sci. 2023; 131:104934.
23. Fazio E, Medica P, Aronica V, Grasso L, Ferlazzo A. Circulating β-endorphin, adrenocorticotrophic hormone and cortisol levels of stallions before and after short road transport: stress effect of different distances. Acta Veter Scand. 2008; 50(1):6.
24. Ayala I, Martos NF, Silvan G, Gutierrez-Panizo C, Clavel JG, Illera JC. Cortisol, adrenocorticotropic hormone, serotonin, adrenaline and noradrenaline serum concentrations in relation to disease and stress in the horse. Res Vet Sci. 2012; 93(1):103–107.
25. Cayado P, Muñoz-Escassi B, Domínguez C, Manley W, Olabarri B, De La Muela MS, et al. Hormone response to training and competition in athletic horses. Equine Vet J. 2006; 38(S36):274–278.
26. Peeters M, Closson C, Beckers J-F, Vandenheede M. Rider and horse salivary cortisol levels during competition and impact on performance. J Equine Vet Sci. 2013; 33(3):155–160.
27. Desmecht D, Linden A, Amory H, Art T, Lekeux P. Relationship of plasma lactate production to cortisol release following completion of different types of sporting events in horses. Vet Res Comms. 1996; 20(4):371–379.
28. Janczarek I, Kędzierski W, Wilk I, Wnuk–Pawlak E, Rakowska A. Comparison of daily heart rate variability in old and young horses: A preliminary study. J Vet Behav. 2020; 38:1–7.
29. Stucke D, Große Ruse M, Lebelt D. Measuring heart rate variability in horses to investigate the autonomic nervous system activity – Pros and cons of different methods. Appl Anim Behav Sci. 2015; 166:1–10.
30. Malik M. Heart rate variability. Curr Opin Cardiol. 1998; 13(1):36–44.
31. Shaffer F, Ginsberg JP. An overview of heart rate variability metrics and norms. Front Publ Health. 2017; 5.
32. Shaffer F, McCraty R, Zerr CL. A healthy heart is not a metronome: an integrative review of the heart's anatomy and heart rate variability. Front Psychol. 2014; 5.
33. von Borell E, Langbein J, Després G, Hansen S, Leterrier C, Marchant J, et al. Heart rate variability as a measure of autonomic regulation of cardiac activity for assessing stress and welfare in farm animals — A review. Physiol Behav. 2007;92(3):293–316.
34. Pasing S, von Lewinski M, Wulf M, Erber R, Aurich C. Influence of semen collection on salivary cortisol release, heart rate, and heart rate variability in stallions. Theriogenol. 2013; 80(3):256–261.
35. van Vollenhoven E, Fletcher L, Page PC, Ganswindt A, Grant CC. Heart rate variability in healthy, adult pony mares during transrectal palpation of the reproductive tract by veterinary students. J Equine Vet Sci. 2017; 58:68–77.
36. Ohmura H, Hobo S, Hiraga A, Jones JH. Changes in heart rate and heart rate variability during transportation of horses by road and air. Amer J Vet Res. 2012; 73(4):515–521.

37. Ohmura H, Hiraga A. Effect of restraint inside the transport vehicle on heart rate and heart rate variability in Thoroughbred horses. J Equine Sci. 2022; 33(1):13–17.
38. Li K, Lai R, Du Y, Ly V, Li D, Lam M, et al. Effects of exercise on heart rate variability by time-domain, frequency-domain and nonlinear analyses in equine athletes [version 1; peer review: 2 approved with reservations]. F1000Research. 2019; 8(147).
39. Ille N, von Lewinski M, Erber R, Wulf M, Aurich J, Möstl E, Aurich C. Effects of the level of experience of horses and their riders on cortisol release, heart rate and heart-rate variability during a jumping course. Anim Welf. 2013; 22(4):457–465.
40. Szabó C, Vizesi Z, Vincze A. Heart rate and heart rate variability of amateur show jumping horses competing on different levels. Animals. 2021; 11(3): 693.
41. Electrophysiology TFotESoCtNASoP. Heart Rate Variability. Circulation. 1996; 93(5):1043–1065.
42. Physick-Sheard PW, Marlin DJ, Thornhill R, Schroter RC. Frequency domain analysis of heart rate variability in horses at rest and during exercise. Equine Vet J. 2000; 32(3):253–262.
43. Claydon VE, Krassioukov AV. Clinical correlates of frequency analyses of cardiovascular control after spinal cord injury. Amer J Physiol-Heart and Circul Physiol. 2008; 294(2):H668–H678.
44. Taylor JA, Carr DL, Myers CW, Eckberg DL. Mechanisms underlying very-low-frequency rr-interval oscillations in humans. Circulation. 1998; 98(6):547–555.
45. Ciccone AB, Siedlik JA, Wecht JM, Deckert JA, Nguyen ND, Weir JP. Reminder: RMSSD and SD1 are identical heart rate variability metrics. Muscle Nerve. 2017; 56(4):674–678.
46. Guzik P, Piskorski J, Krauze T, Schneider R, Wesseling KH, Wykretowicz A, Wysocki H. Correlations between the Poincaré plot and conventional heart rate variability parameters assessed during paced breathing. J Physiol Sci. 2007; 57(1):63–71.
47. Schmidt A, Möstl E, Wehnert C, Aurich J, Müller J, Aurich C. Cortisol release and heart rate variability in horses during road transport. Horm Behav. 2010; 57(2):209–215.
48. von Lewinski M, Biau S, Erber R, Ille N, Aurich J, Faure J-M, et al. Cortisol release, heart rate and heart rate variability in the horse and its rider: Different responses to training and performance. Vet J. 2013; 197(2):229–232.
49. Ispirlidis I, Fatouros IG, Jamurtas AZ, Nikolaidis MG, Michailidis I, Douroudos I, et al. Time-course of changes in inflammatory and performance responses following a soccer game. Clin J Sport Med. 2008; 18(5).
50. Coelho DB, Mortimer LÁ, Condessa LA, Morandi RF, Oliveira BM, Marins JCB, et al. Intensity of real competitive soccer matches and differences among player positions. Revista Brasileira de Cineantropometria & Desempenho Humano. 2011; 13.
51. Slimani M, Nikolaidis PT. Anthropometric and physiological characteristics of male soccer players according to their competitive level, playing position and age group: a systematic review. J Sports Med Phys Fitness. 2017; 59(1):141–163.
52. Lipponen JA, Tarvainen MP. A robust algorithm for heart rate variability time series artefact correction using novel beat classification. J Med Eng Technol. 2019; 43(3):173–181.
53. Bloomfield J, Polman R, O'Donoghue P. Physical demands of different positions in fa premier league soccer. J Sports Sci Med. 2007; 6(1):63–70.
54. Villas-Boas JD, Dias DPM, de Almeida FQ, de Medeiros MA. Different equestrian sports horses' reactivity to startle. J Vet Behav. 2022; 57:16–23.
55. Irvine CHG, Alexander SL. Factors affecting the circadian rhythm in plasma cortisol concentrations in the horse. Domest Anim Endocrinol. 1994; 11(2):227–238.

56. Nagata S, Takeda F, Kurosawa M, Mima K, Hiraga A, Kai M, Taya K. Plasma adrenocorticotropin, cortisol and catecholamines response to various exercises. Equine Vet J. 1999; 31(S30):570–574.

57. Bohák Z, Szabó F, Beckers JF, Melo de Sousa N, Kutasi O, Nagy K, Szenci O. Monitoring the circadian rhythm of serum and salivary cortisol concentrations in the horse. Domest Anim Endocrinol. 2013; 45(1):38–42.

58. Contreras-Aguilar MD, Lamy E, Escribano D, Cerón JJ, Tecles F, Quiles AJ, Hevia ML. Changes in salivary analytes of horses due to circadian rhythm and season: a pilot study. Animals. 2020; 10(9).

59. Joongpan W, Tongsangiam P, Poochipakorn C, Charoenchanikran P, Chanda M. Influence of full-time housing in vector-protected facilities on equine cortisol levels, heart rate, and behavior during the African horse sickness outbreak in Thailand. J Appl Anim Welf Sci. 2023; 26(1):52–67.

60. Poochipakorn C, Joongpan W, Tongsangiam P, Phooseerit A, Leelahapongsathon K, Chanda M. The impact of strategic ventilation adjustments on stress responses in horses housed full-time in a vector-protected barn during the African horse sickness outbreak in Thailand. Anim Welf. 2023; 32:e19.

61. Teixeira-Neto AR, Ferraz GdC, Moscardini ARC, Balsamão G, Souza JCF, Queiroz-Neto AD. Alterations in muscular enzymes of horses competing long-distance endurance rides under tropical climate. Arquivo Brasileiro de Medicina Veterinária e Zootecnia. 2008; 60:543–549.

62. Bayly WM, Kingston JK, Brown JA, Keegan RD, Greene SA, Sides RH. Changes in arterial, mixed venous and intraerythrocytic concentrations of ions in supramaximally exercising horses. Equine Vet J. 2006; 38(S36):294–297.

63. Kearns CF, McKeever KH, John-Alder H, Abe T, Brechue WF. Relationship between body composition, blood volume and maximal oxygen uptake. Equine Vet J. 2002; 34(S34):485–490.

64. Pedersen BK, Hoffman-Goetz L. Exercise and the immune system: regulation, integration, and adaptation. physiological reviews. 2000; 80(3):1055–1081.

65. McCarthy DA, Macdonald I, Grant M, Marbut M, Watling M, Nicholson S, et al. Studies on the immediate and delayed leucocytosis elicited by brief (30-min) strenuous exercise. Eur J Appl Physiol Occup Physiol. 1992; 64(6):513–517.

66. Krumrych W, Gołda R, Gołyński M, Markiewicz H, Buzała M. Effect of physical exercise on cortisol concentration and neutrophil oxygen metabolism in peripheral blood of horses. Annals Anim Sci. 2018; 18(1):53–68.

67. Wang J, Liu S, Li G, Xiao J. Exercise regulates the immune system. In: Xiao J, editor. Physical exercise for human health. Singapore: Springer Singapore; 2020. pp. 395–408.

68. Peake JM. Exercise-induced alterations in neutrophil degranulation and respiratory burst activity: possible mechanisms of action. Exerc Immunol Rev. 2002; 8:49–100.

69. Robson PJ, Blannin AK, Walsh NP, Castell LM, Gleeson M. Effects of exercise intensity, duration and recovery on in vitro neutrophil function in male athletes. Int J Sports Med. 1999; 20(02):128–130.

70. Mair TS, Sherlock CE, Boden LA. Serum cortisol concentrations in horses with colic. Vet J. 2014; 201(3):370–377.

71. Lorello O, Ramseyer A, Burger D, Gerber V, Bruckmaier RM, van der Kolk JH, Navas de Solis C. Repeated measurements of markers of autonomic tone over a training season in eventing horses. J Equine Vet Sci. 2017; 53:38–44.

72. Akselrod S, Gordon D, Ubel FA, Shannon DC, Berger AC, Cohen RJ. Power spectrum analysis of heart rate fluctuation: a quantitative probe of beat-to-beat cardiovascular control. Science. 1981; 213(4504):220–222.

Click here to access/download;Figure;Fig. 1 hadicap speed and distance-01.tif

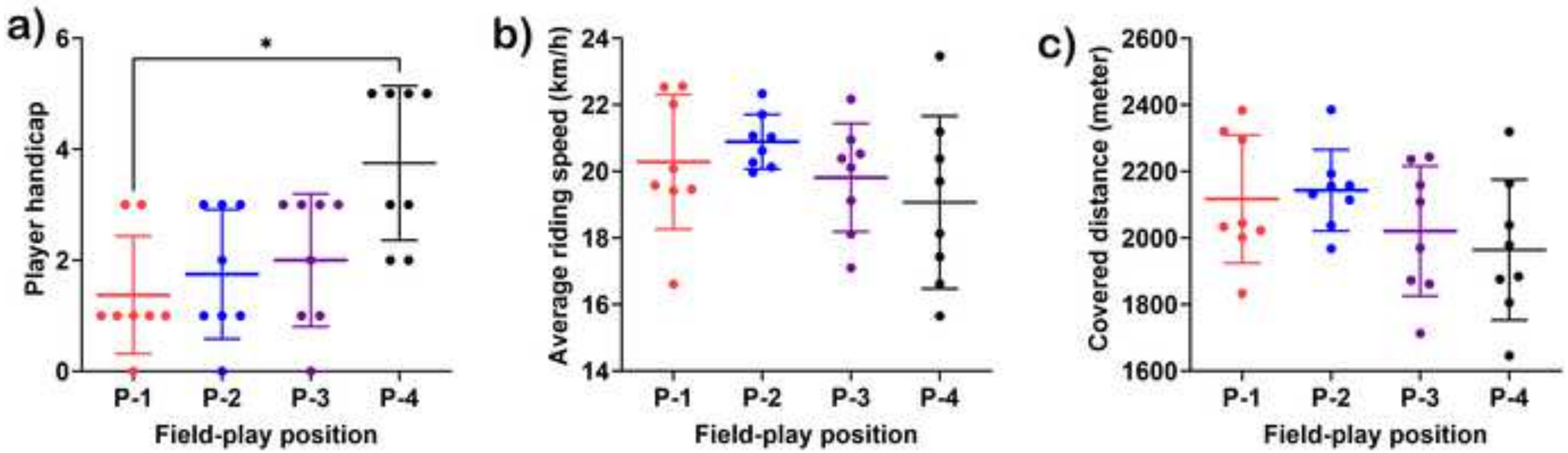

Figure 2

Click here to access/download;Figure;Fig. 2 HR Zone each position-01.tif

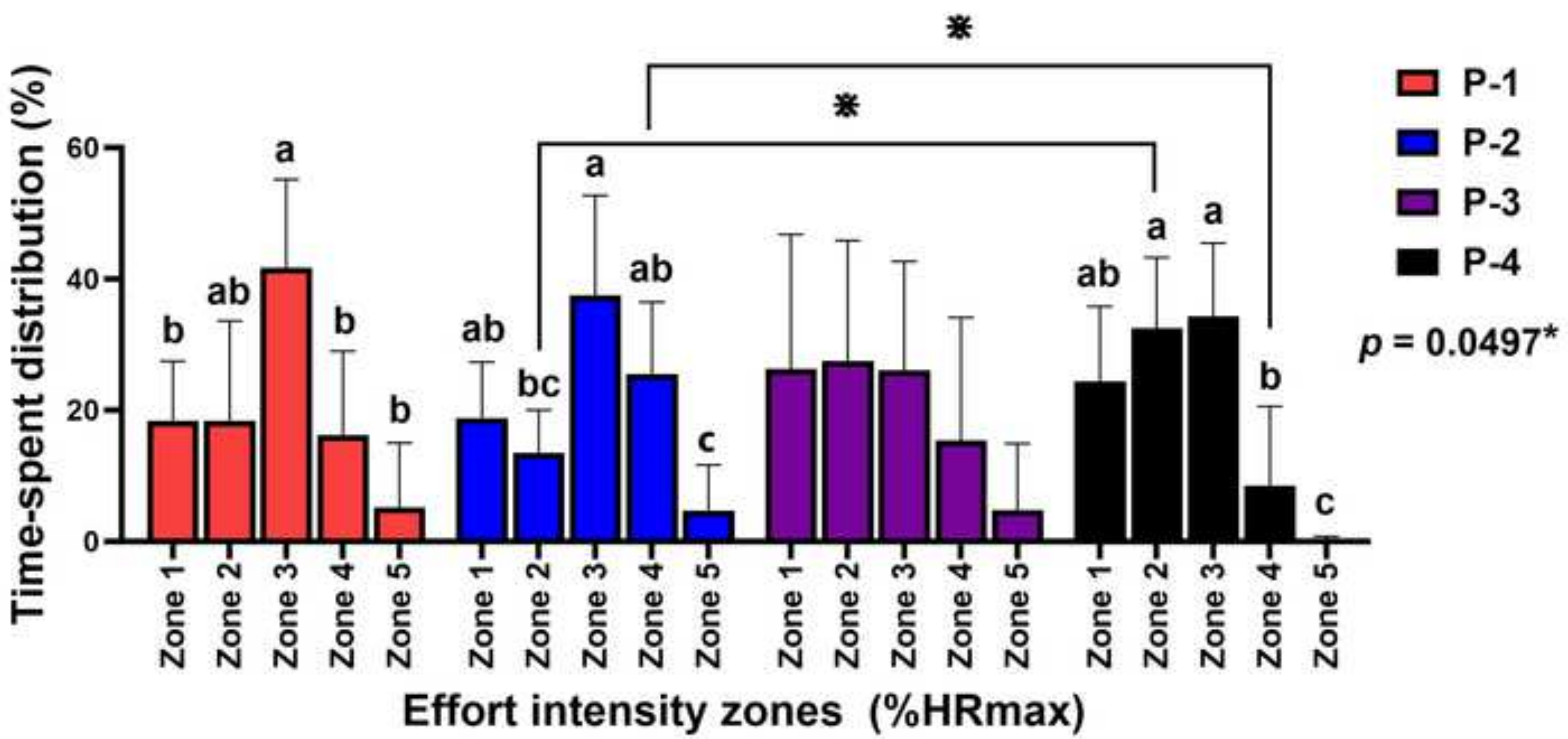

Figure 3 Click here to access/download;Figure;Fig. 3 HR and RR-01.tif

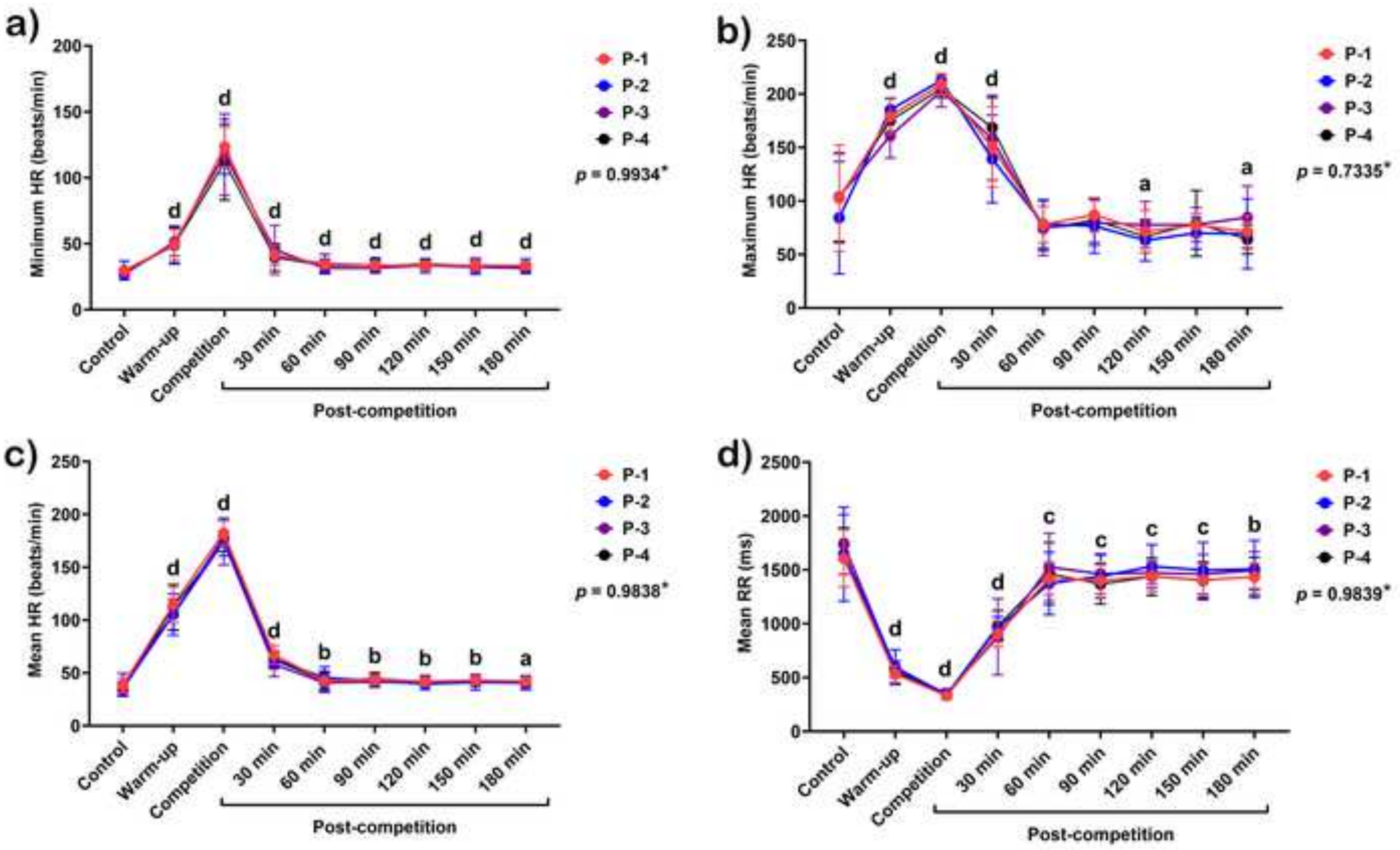

Figure 4

Click here to access/download;Figure;Fig. 4 SNDNN RMSSD pNN50 and SI-01.tif

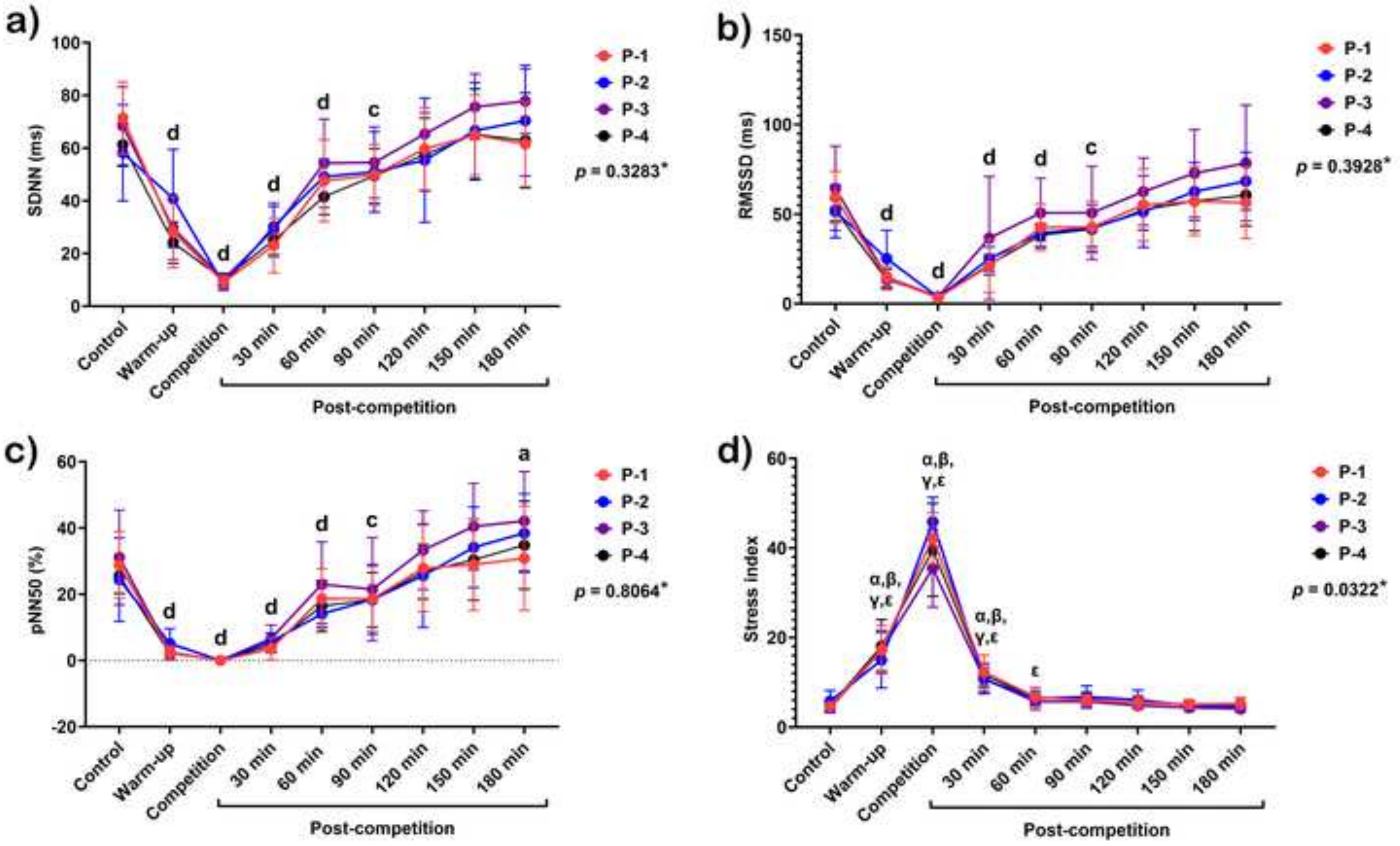

Figure 5

Click here to access/download;Figure;Fig. 5 TINN RTI DC DCmod-01.tif

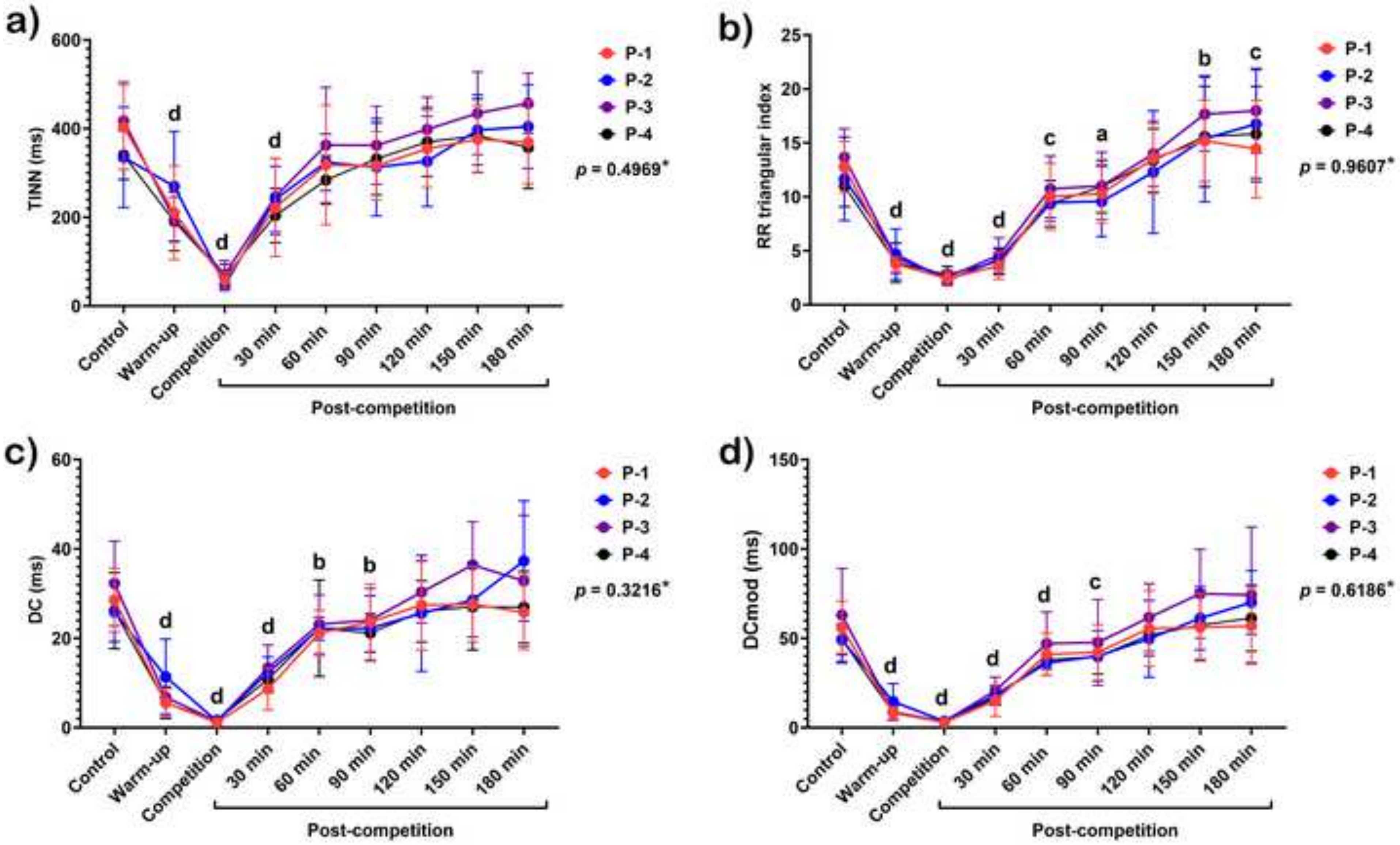

Figure 6

Click here to access/download;Figure;Fig. 6 VLF LF HF LF-HF ratio-01.tif

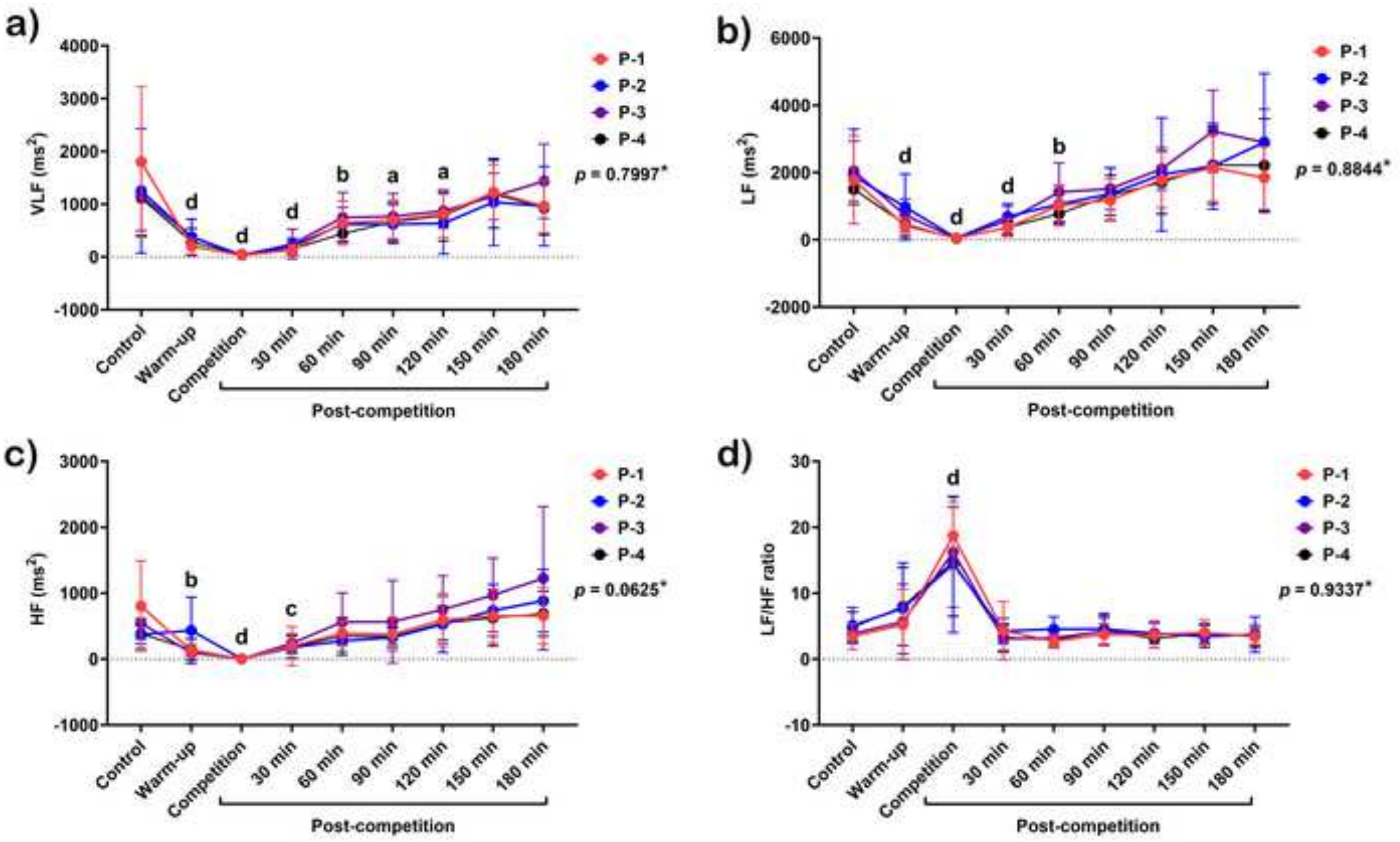

Figure 7

Click here to access/download;Figure;Fig. 7 total power and frequency percent-01.tif

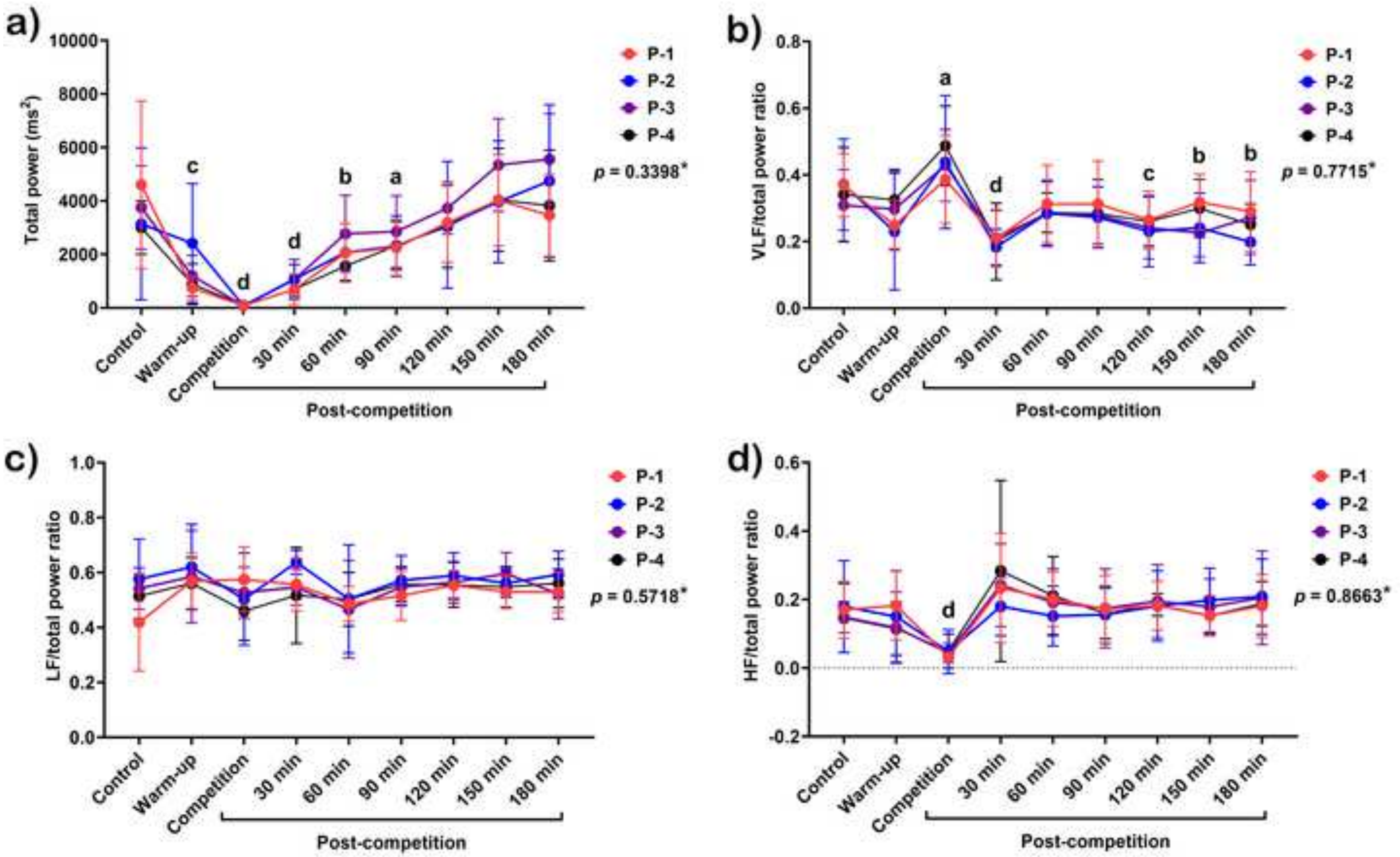

Figure 8

Click here to access/download;Figure;Fig. 8 RESP SD1 and 2 and SD2-SD1 ratio-01.tif

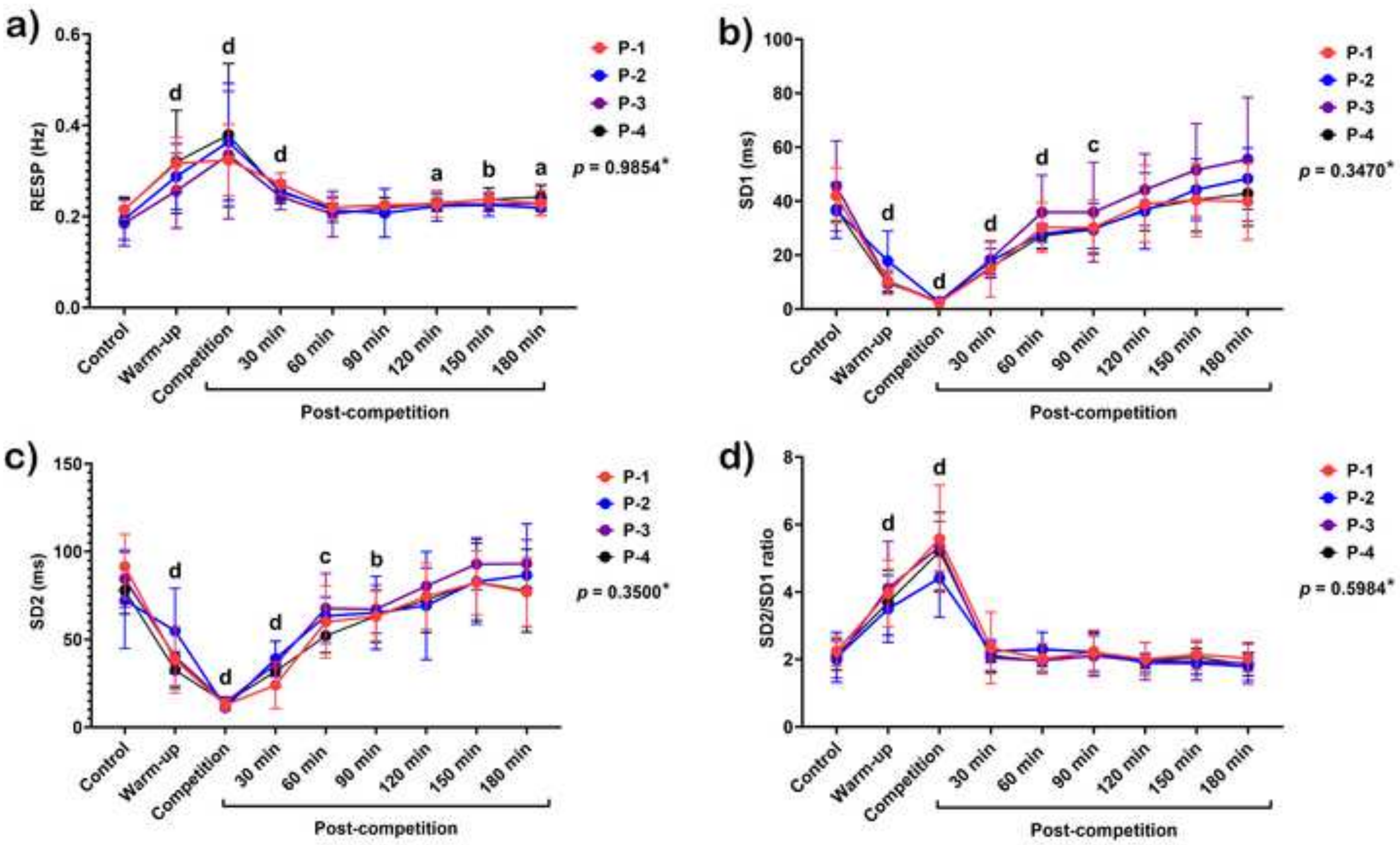

Figure 9

Click here to access/download;Figure;Fig. 9 ANS indexes-01.tif

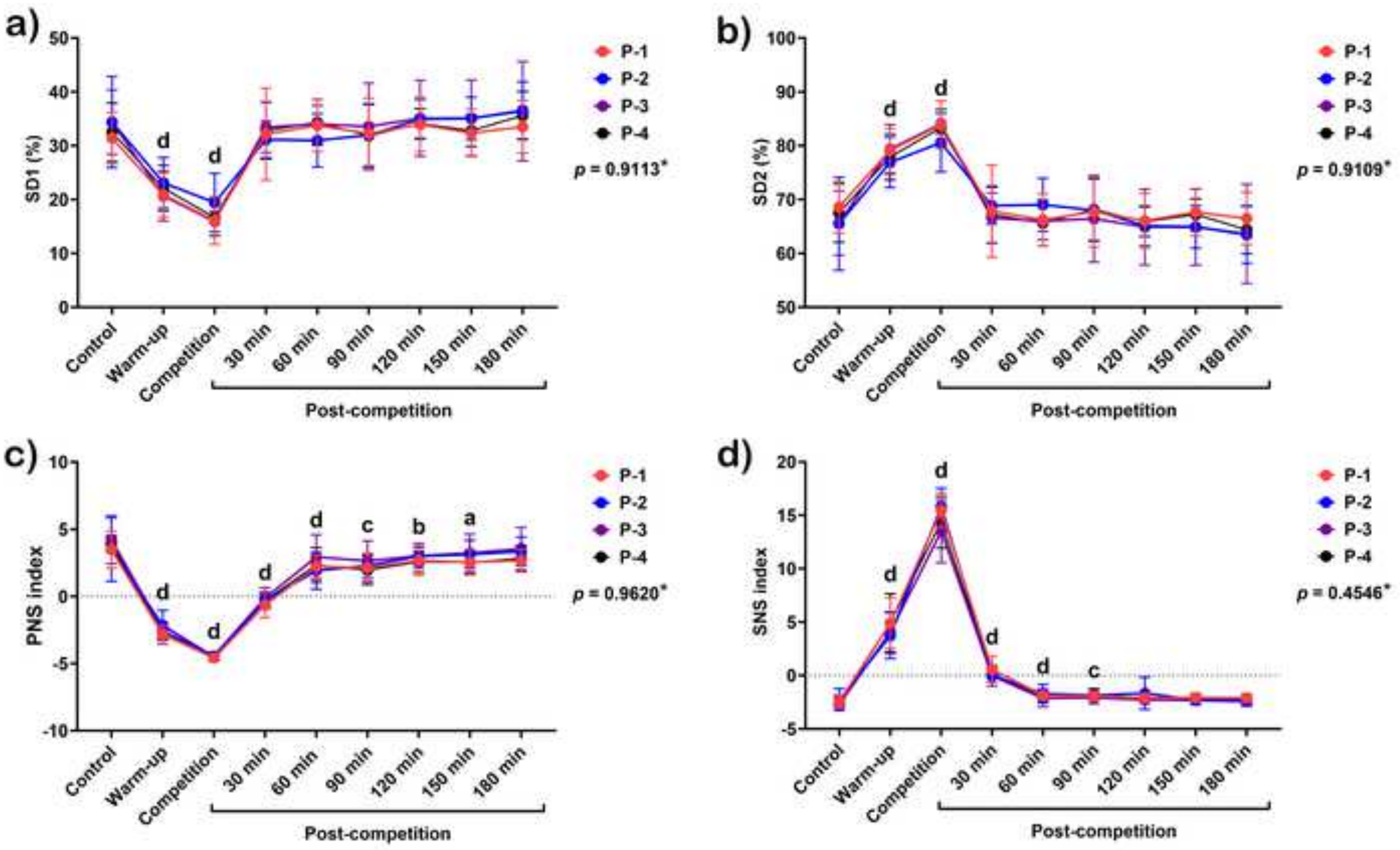

Supplementary File S1

Click here to access/download
Supporting Information
Fig. S1 MATLAB file.docx

Click here to access/download
Supporting Information
certificate of English proofreading.pdf
